# METTL1-mediated tRNA m^7^G methylation and translational dysfunction restricts breast cancer tumorigenesis by fueling cell cycle blockade

**DOI:** 10.1186/s13046-024-03076-x

**Published:** 2024-05-31

**Authors:** Dan Du, Mingxia Zhou, Chenxi Ju, Jie Yin, Chang Wang, Xinyu Xu, Yunqing Yang, Yun Li, Le Cui, Zhengyang Wang, Yuqing Lei, Hongle Li, Fucheng He, Jing He

**Affiliations:** 1 Department of Medical Laboratory, The First Affiliated Hospital of Zhengzhou University, Zhengzhou, 450052 China; 2 Department of Gastroenterology, The First Affiliated Hospital of Zhengzhou University, Zhengzhou, 450052 China; 3 Department of Oncology, The First Affiliated Hospital of Zhengzhou University, Zhengzhou, 450052 China; 4 Department of Breast Surgery, The First Affiliated Hospital of Zhengzhou University, Zhengzhou, 450052 China; 5 Department of Pathology, The First Affiliated Hospital of Zhengzhou University, Zhengzhou, 450052 China; 6 Department of Molecular Pathology, The Affiliated Cancer Hospital of Zhengzhou University, Zhengzhou, 450008 China

**Keywords:** METTL1, Breast cancer, CDK4/6, GADD45A, Cell cycle

## Abstract

**Background:**

RNA modifications of transfer RNAs (tRNAs) are critical for tRNA function. Growing evidence has revealed that tRNA modifications are related to various disease processes, including malignant tumors. However, the biological functions of methyltransferase-like 1 (METTL1)-regulated m^7^G tRNA modifications in breast cancer (BC) remain largely obscure.

**Methods:**

The biological role of METTL1 in BC progression were examined by cellular loss- and gain-of-function tests and xenograft models both in vitro and in vivo. To investigate the change of m^7^G tRNA modification and mRNA translation efficiency in BC, m^7^G-methylated tRNA immunoprecipitation sequencing (m^7^G tRNA MeRIP-seq), Ribosome profiling sequencing (Ribo-seq), and polysome-associated mRNA sequencing were performed. Rescue assays were conducted to decipher the underlying molecular mechanisms.

**Results:**

The tRNA m^7^G methyltransferase complex components METTL1 and WD repeat domain 4 (WDR4) were down-regulated in BC tissues at both the mRNA and protein levels. Functionally, METTL1 inhibited BC cell proliferation, and cell cycle progression, relying on its enzymatic activity. Mechanistically, METTL1 increased m^7^G levels of 19 tRNAs to modulate the translation of growth arrest and DNA damage 45 alpha (GADD45A) and retinoblastoma protein 1 (RB1) in a codon-dependent manner associated with m^7^G. Furthermore, in vivo experiments showed that overexpression of METTL1 enhanced the anti-tumor effectiveness of abemaciclib, a cyclin-dependent kinases 4 and 6 (CDK4/6) inhibitor.

**Conclusion:**

Our study uncovered the crucial tumor-suppressive role of METTL1-mediated tRNA m^7^G modification in BC by promoting the translation of GADD45A and RB1 mRNAs, selectively blocking the G2/M phase of the cell cycle. These findings also provided a promising strategy for improving the therapeutic benefits of CDK4/6 inhibitors in the treatment of BC patients.

**Supplementary Information:**

The online version contains supplementary material available at 10.1186/s13046-024-03076-x.

## Introduction

The incidence rates of breast cancer (BC) have increased steadily over the past four decades, with an annual growth rate of 0.5%. BC is the second leading cause of cancer incidence worldwide, accounting for 11.6% of all cancer cases. It ranks fourth in terms of cancer mortality, resulting in over 665,700 deaths per year globally [1, 2]. Thankfully, advancements in early detection and multiple therapy approaches have substantially improved the overall survival of BC [3]. However, the high recurrence rate and growing resistance to current therapeutics necessitate scientific exploration of reliable biomarkers and promising therapeutic targets for BC treatment.

Epigenetic modifications play a crucial role in cancer development and progression [4]. To date, researchers have identified at least 170 distinct kinds of post-transcriptional RNA modifications that encompass various RNA molecules [5]. Among them, N^7^-methylguanosine (m^7^G), mediated by a complex consisting of methyltransferase-like 1 (METTL1) and WD repeat domain 4 (WDR4) in humans, is one of the most prevalent tRNA modifications found in the variable loop at position 46 [6]. Recent studies have demonstrated an association between dysregulated METTL1 expression and poor prognosis as well as malignant phenotypes in multiple cancer types [7, 8]. However, the expression, function, and underlying mechanisms of METTL1-mediated m^7^G tRNA modification in BC remain largely unexplored.

It is widely recognized that uncontrolled cell proliferation, genomic instability, and cancer initiation are closely related to abnormal activation and progression of the cell cycle [9]. Deregulation of the cell cycle is a universal hallmark of cancer and is considered a potential mechanism for inhibiting tumor development or enhancing drug sensitivity [10]. G1 cell cycle arrest is a crucial feature of senescent cells, while the G2/M phase cell block is the main cause of cell death [11, 12]. Thus, targeting cell cycle regulators to induce cell cycle arrest has emerged as a promising anti-cancer strategy [13]. Among the stress sensor family GADD45, growth arrest and DNA damage 45 alpha (GADD45A) is a stress-inducible gene, regulated by p53 and BRCA1, functioning in the regulation of a variety of cellular functions such as DNA repair, cell cycle control, and genomic stability [14]. It plays a critical role in the cellular response to DNA damage agents, leading to cell cycle arrest, DNA repair, or apoptosis [15]. The retinoblastoma protein 1 (RB1), a notable tumor suppressor, plays a pivotal role in cell-cycle checkpoints and limits cell cycle progression [16]. GADD45A and RB1 tightly modulate the cyclin-dependent kinases (CDKs) activity and cyclin levels [17, 18]. Abemaciclib, an orally bioavailable and highly selective competitive inhibitor of CDK4/6, has been approved for treating refractory hormone receptor-positive (HR+), human epidermal growth factor receptor 2-negative (HER2-) metastatic BC [19]. However, the clinical application of CDK4/6 inhibitors faces challenges, such as developing novel combination regimens and understanding the biological mechanisms underlying CDK4/6 inhibitor resistance.

In this study, we detailedly investigated the action and molecular mechanism of METTL1-mediated m^7^G tRNA modification in BC pathogenesis. Our findings uncovered the essential role of METTL1 in regulating BC malignant transformation and provided a molecular basis for understanding tRNA-mediated codon-biased translation in BC.

## Materials and methods

### Clinical specimens

Human BC tissues and corresponding adjacent normal tissues were collected from patients without preoperative chemotherapy or radiotherapy from the First Affiliated Hospital of Zhengzhou University. Adjacent non-tumor specimens were obtained at a standardized distance of 5 cm from the BC tumor tissues. The tissues were promptly collected post-surgery and stored at -80 °C until use. The characteristics of sixty-six pairs of BC patients are shown in Supplementary Table S1. Written informed consent was obtained from each patient in the cohort.

### Cell culture

Human BC cell lines (MDA-MB-415, T47D, MCF-7, MDA-MB-231, ZR-75-1, BT-549, MDA-MB-468, BT-474, AU565) and the human mammary epithelial cell line (MCF10A) were obtained from the American Type Culture Collection (ATCC, USA). These cell lines were cultured in DMEM or RPMI 1640 media (BDBIO, HangZhou, China) supplemented with 10% fetal bovine serum (FBS, VivaCell, China) and 1% penicillin-streptomycin (Solarbio, China). The cell plates were incubated at 37 °C with 5% CO_2_ under saturated humidity conditions.

### Quantitative reverse transcriptase polymerase chain reaction (qRT-PCR)

Total RNA from cells and tissues was extracted using Trizol (Takara, Japan) following the manufacturer’s instructions. cDNA synthesis was performed using the PrimeScript™ RT Master Mix (Takara, Japan). qRT-PCR was conducted using the Applied Biosystems 7500 Real-Time PCR System (Applied Biosystems, USA) and Hieff qPCR SYBR Green Master Mix (YEASEN, China). The relative mRNA expression levels were determined using the 2^–ΔΔCt^ method with GAPDH as the reference gene. The primers used for qRT-PCR are presented in Supplementary Table S2.

### RNA interference and lentivirus-mediated infection

Small interfering RNA (siRNA) targeting METTL1 and GADD45A and a corresponding control (si-NC) were obtained from RiboBio (Guangzhou, China). Transfection was performed using the RiboFECT CP Transfection Kit (Guangzhou, China). The coding sequence region of METTL1 (NM_005371.6) was amplified and cloned into the pCDH-CMV-MCS-EF1-Puro lentiviral vector. A catalytically inactive mutant of METTL1 (aa160-163, LFPD to AFPA) and GADD45A mutants were generated by GENEWIZ (Suzhou, China) using the same vector as the wild-type METTL1 [20]. Plasmid transfection into 293T cells was carried out with packaging vectors pMD2.G and psPAX2. Forty-eight hours after transfection, we harvested the viral supernatant to be filtered through a 0.45 μm filter, and used them to infect target cells at 50% confluence. Puromycin (TargetMol, USA) was used to select stably transfected cells. Transfection efficiency was assessed by qRT-PCR or western blotting. The sequence of the siRNA is provided in Supplementary Table S3.

### Western blotting

Cells were lysed with RIPA buffer (Epizyme, China) supplemented with protease inhibitors (Beyotime, China). Protein concentrations were determined using a BCA protein assay kit (Novoprotein, China) and normalized. Protein lysates were separated by sodium dodecyl sulfate-polyacrylamide gel electrophoresis (SDS-PAGE) and transferred onto PVDF membranes (Millipore, USA). Band visualization was achieved using an ECL chemiluminescent reagent (Affinibody, China). Antibodies used are listed in Supplementary Table S4.

### Immunohistochemistry (IHC) staining

IHC staining was performed following previously described methods [21]. BC tissue sections were deparaffinized, dehydrated, subjected to antigen retrieval, blocked, and incubated with primary antibodies. Diaminobenzidine detection was used for staining. Image J software was utilized for IHC analysis. The protein levels of METTL1, WDR4, and Ki67 were quantified by calculating the mean density of positive staining in five randomly selected fields.

### Functional assays in vitro

Colony formation assays were conducted by seeding MDA-MB-231 or MCF-7 cells at a density of 1000 cells per well in six-well plates with fresh medium, followed by incubation at 37 °C. After 2 weeks, the number of colonies was determined by staining with 0.5% crystal violet. Cell viability assays involved seeding 2000 transfected cells per well in 96-well plates with fresh medium. Cell viabilities were assessed at 0, 24, 48, 72, and 96 h using the Cell Counting Kit-8 (Servicebio, Wuhan, China) according to the manufacturer’s instructions. The EdU cell proliferation assay was performed using the Cell-Light EdU Apollo567 In Vitro Kit (RiboBio, Guangzhou, China). Transfected cells were stained with 50 µM EdU reagent for 2 h, fixed with 4% paraformaldehyde for 30 min, and subsequently stained with Apollo reaction solution and DAPI solution. The fluorescence signal was observed under an inverted fluorescence microscope, and the proportion of EdU-positive cells was analyzed using Image J. Cell migration and invasion were assessed using transwell and wound-healing assays. For transwell assays, a total of 2 × 10^5^ transfected cells resuspended in 200 µL serum-free fresh medium were added to the upper chamber with or without Matrigel (NEST Biotechnology). After 24–48 h, the cells on the outer surface of the membrane were fixed with 4% paraformaldehyde and counted after staining with crystal violet to analyze cell migration and invasion. Wound-healing assays involved culturing transfected cells in 6-well plates until reaching 80% confluence, then creating a gap by scratching with a 200 µL pipette tip. Cells were cultured in a serum-free medium, and photographs were taken under an inverted microscope at 0 and 24 h after the scratch. The proportion of the wound-healing area was analyzed using Image J.

### Cell cycle analysis

Cells were harvested, washed with cold PBS, fixed in 70% ethanol at 4 °C overnight, and then incubated with RNase and the DNA-interacting dye propidium iodide (PI) for 30 min at 37 °C. Cell cycle analysis was performed using a flow cytometer (BD Accuri C6, CA, USA), and the results were further examined and graphically exported using FlowJoTM v.10 Software BD Biosciences (Franklin Lakes, NJ, USA).

### TdT-mediated dUTP Nick-End labeling (TUNEL) staining

Cell apoptosis was assessed using the TUNEL Cell Apoptosis Assay Kit (RiboBio, Guangzhou, China). Transfected cells were fixed with paraformaldehyde and then incubated with TUNEL solution for 30 min. After staining with DAPI for 30 min, cell images were captured using a fluorescence microscope. The number of TUNEL-positive cells was analyzed using Image J software to determine cell apoptosis.

### m^7^G tRNA MeRIP-Seq

The technical service for m^7^G tRNA MeRIP-Seq was provided by Cloudseq Biotech Inc. (Shanghai, China). In brief, fragmented RNA was incubated with anti-m^7^G polyclonal antibody (Synaptic Systems, 202003) in IPP buffer at 4 °C for 2 h. The mixture underwent further immunoprecipitation by incubating with Protein A beads (Thermo Fisher) at 4 °C for an additional 2 h. The purified RNA was size-selected for the fraction of RNA less than 200 nt using the MirVana Isolation Kit (Invitrogen). Subsequently, the RNAs were then de-aminoacylated in 1 mM EDTA and 0.1 M Tris-HCl for 30 min at 37 °C. tRNA libraries were constructed following the instructions of the TruSeq Small RNA Preparation Kit (Illumina). All libraries were size-selected (170–210 bp) prior to sequencing, which was performed on a HiSeq platform (Illumina).

### Polysome profiling

BC cells were cultured with cold PBS with 100 µg/mL cycloheximide for 3 min, and subsequently lysed with polysome lysis buffer for 10 min on ice. After centrifugation of the cell lysate (13,000 g, 10 min, 4 ℃), the supernatant was carefully layered on top of sucrose gradients and then centrifuged at 36,000 rpm for 3 h at 4 ℃. Samples obtained after ultracentrifugation were immediately fractionated at a speed of 75 mL/min using the Brandel BR-188 Density Gradient Fractionation Device. OD254 values were continuously monitored and recorded during fractionation.

### Puromycin intake assay

To measure the rate of new protein translation, cells were incubated with 1 µM puromycin for 30 min at 37 ℃. After incubation, cells were lysed to extract proteins, and western blotting was performed using an anti-puromycin antibody (Kerafast) to detect the level of puromycin incorporation.

### Ribosome profiling sequencing (Ribo-seq)

Cycloheximide (Sigma, USA) was added to the cell culture medium at a final concentration of 100 µg/mL and thoroughly mixed with the transfected cells. After incubating for 2 min, the Ribo-seq technique was performed by Hangzhou Kaitai Biotechnology Co. Ltd. Briefly, the cells were triturated to collect the supernatant, which was then treated with 10 µL of RNase I (NEB, MA, USA) and 6 µL of DNase I (NEB, Ipswich, MA, USA) to degrade RNA fragments without ribosome coverage. The ribosomes were subsequently removed. Small fragments of approximately 30 bp, known as ribosomal footprints (Rfs), which represented the translated RNA protected by ribosomes, were sequenced using Illumina HiSeqTM X10 by Gene Denovo Biotechnology Co. (Guangzhou, China). The FPKM values were calculated using RSEM software to determine gene translation levels. Differentially translated genes (DTGs) were identified by applying FDR < 0.05 and |log2FC| > 1, enabling analysis of differential translation between groups.

### Polyribosome-associated mRNA qPCR

To assess the translation efficiencies (TEs) of specific genes, polyribosome-associated mRNA qPCR was conducted [22]. Cells were rinsed with pre-cooled PBS containing 100 µg/mL cycloheximide. Cell lysates were incubated on ice for 30 min using cell lysis buffer and then centrifuged (16,200 g, 10 min, 4 °C). The supernatants were divided into two fractions: 10% of the supernatant was saved as the input control for subsequent qRT-PCR, and the remaining supernatants were subjected to ultracentrifugation in a SW32 rotor (Beckman Coulter) to collect polysomes. RNA was isolated from the polysome fractions using TRIzol Reagent (Takara, Japan), followed by qRT-PCR analysis of the indicated genes.

### Animal models

All animal experimental programs were conducted in compliance with the Institutional Animal Care and Use Committee of the First Affiliated Hospital of Zhengzhou University. Female BALB/c nude mice (5 weeks old) were purchased from Vital River Laboratory Animal Technology Co. Ltd (Beijing, China). For the in vivo xenograft model, mice were randomly assigned to three groups (*n* = 6). Stably transfected MCF-7 cells (oe-NC, oe-M1, oe-Mut), as described earlier, were dispersed to form single-cell suspensions, and each nude mouse was subcutaneously injected with 4 × 10^6^ cells. After 3 weeks from the tumor inoculation, the mice were sacrificed. MCF-7 cells (4 × 10^6^) were subcutaneously injected into nude mice. Ten days after inoculation, when the tumor volume reached close to 100 mm^3^, the mice were randomized into four groups (*n* = 6): control (LV-NC + DMSO), oe-METTL1 (LV-M1 + DMSO), abemaciclib (LV-NC + Abe), and oe-METTL1 + abemaciclib (LV-M1 + Abe). The control lentivirus (LV-NC) and METTL1 lentivirus (LV-METTL1) were obtained from Obio Technology Company (Shanghai, China). The mice were injected with the indicated lentiviruses via multipoint intratumoral injection every week for three doses. Abemaciclib was administered orally at 80 mg/kg (once a week for three doses) using a gavage injection with a volume of 0.2 mL/dose. Initially, abemaciclib was dissolved in DMSO and then diluted in water with 0.4% carboxymethylcellulose (Merck Millipore) and 0.2% Tween-80 (Sigma). Throughout the drug treatment period, tumor volume (length × width^2^ × 0.5/mm^3^) was measured every 3 days, and tumors were collected after 3 weeks of treatment.

### Statistical analysis

Data from all experiments are expressed as mean ± standard deviation (SD). Statistical analyses were performed with SPSS 20.0 (Chicago, USA) and GraphPad Prism 9.0 (La Jolla, CA, USA). Paired or unpaired Student’s t-test was used to determine statistical expression differences between the two groups. Pearson correlation analysis was performed to evaluate the correlation between METTL1 and WDR4, GADD45A, and RB1. Experimental data used for statistical analysis came from at least three representative independent experiments. Significant differences were shown by *P < 0.05, **P < 0.01, ***P < 0.001, and ns., not significant.

## Results

### METTL1/WDR4 are downregulated in BCs

**Fig. 1:**
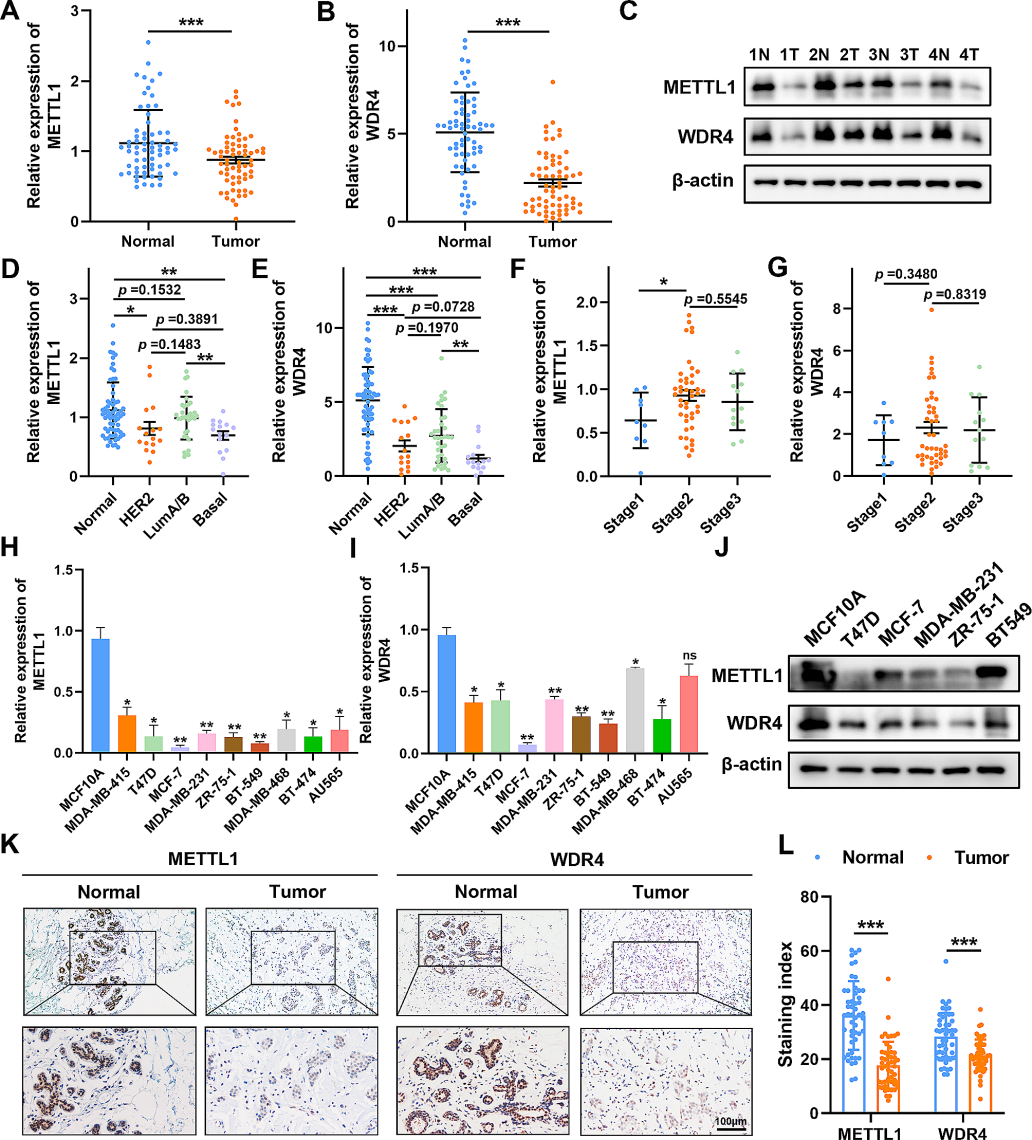
Downregulation of METTL1 and WDR4 in BC patients. (**A**-**B**) qRT-PCR analysis revealed a significant decrease in the expression of METTL1 (**A**) and WDR4 (**B**) in BC tissues compared to adjacent non-tumor tissues. (**C**) Western blotting of METTL1 and WDR4 protein levels in four pairs of human BC specimens. N: Normal, T: Tumor. (**D**-**G**) Stratification of METTL1 and WDR4 mRNA expression levels in BC tissues based on different subtypes (**D**-**E**) and tumor stages (**F**-**G**). (**H**-**J**) qRT-PCR (**H**-**I**) and western blotting (**J**) of METTL1 and WDR4 mRNA and protein levels in BC cell lines. The normal human mammary epithelial cells, MCF10A, were used as a normal control. (K-L) Representative images (**K**) and quantification (**L**) of METTL1 and WDR4 immunostaining in BC specimens and adjacent normal tissues. Scale bar = 100 μm. The data are presented as mean ± SD. *P < 0.05, **P < 0.01, ***P < 0.001

In order to clarify the potential role of the m^7^G tRNA modification in BC, we initially assessed the mRNA expression of METTL1 and WDR4, which constitute the m^7^G tRNA methyltransferase complex. Using qRT-PCR, we examined 66 paired primary BC specimens and observed robust downregulation of METTL1 and WDR4 in BC samples compared to normal breast tissues (Fig. 1A-B). To evaluate the diagnostic utility of METTL1 and WDR4 as non-invasive biomarkers for BC detection, we employed Receiver operating characteristic (ROC) curves, and the area under the ROC curve (AUC) was calculated. The AUC for METTL1 was 0.6416, while the AUC for WDR4 was 0.8510, suggesting the potential of these markers for BC detection (*P* < 0.005, Supplementary Fig. S1A). Moreover, a strong correlation between METTL1 and WDR4 expression levels was evident in BC tissues (Supplementary Fig. S1B). Western blotting analysis further confirmed the decreased protein expression of METTL1 and WDR4 in BC tissues (Fig. 1C). Subsequent examination of METTL1 and WDR4 mRNA transcripts in different BC subtypes revealed slightly lower relative levels in HER2-positive or triple-negative subtypes compared to luminal A and B subtypes, as well as normal breast tissues (Fig. 1D-E). Statistical analysis demonstrated some associations between pathologic stage and METTL1 expression and a non-significant relationship with WDR4. However, no obvious differences in the expression levels of METTL1 and WDR4 were observed with respect to tumor stage and lymph node metastasis (Fig. 1F-G; Supplementary Fig. S1C-F). The expression of METTL1 and WDR4 was analyzed based on the ER, PgR, and ERBB2 status. Both METTL1 and WDR4 had higher expression levels in ER-positive BC patients, and WDR4 was also expressed at appreciably higher levels in PR-positive patients (Supplementary Fig. S1G-L). Consistent with the findings, qRT-PCR and western blotting analysis indicated that METTL1 and WDR4 were down-regulated in BC cell lines compared to immortalized epithelial breast cells MCF10A (Fig. 1H-J). Additionally, IHC staining of 50 BC clinical specimens revealed predominant nuclear expression of both METTL1 and WDR4. Quantitative analysis further confirmed a significant reduction in protein expression levels of METTL1 and WDR4 in BC compared to adjacent normal tissues (Fig. 1K-L).

### METTL1 inhibition drives BC cell proliferation and migration

**Fig. 2:**
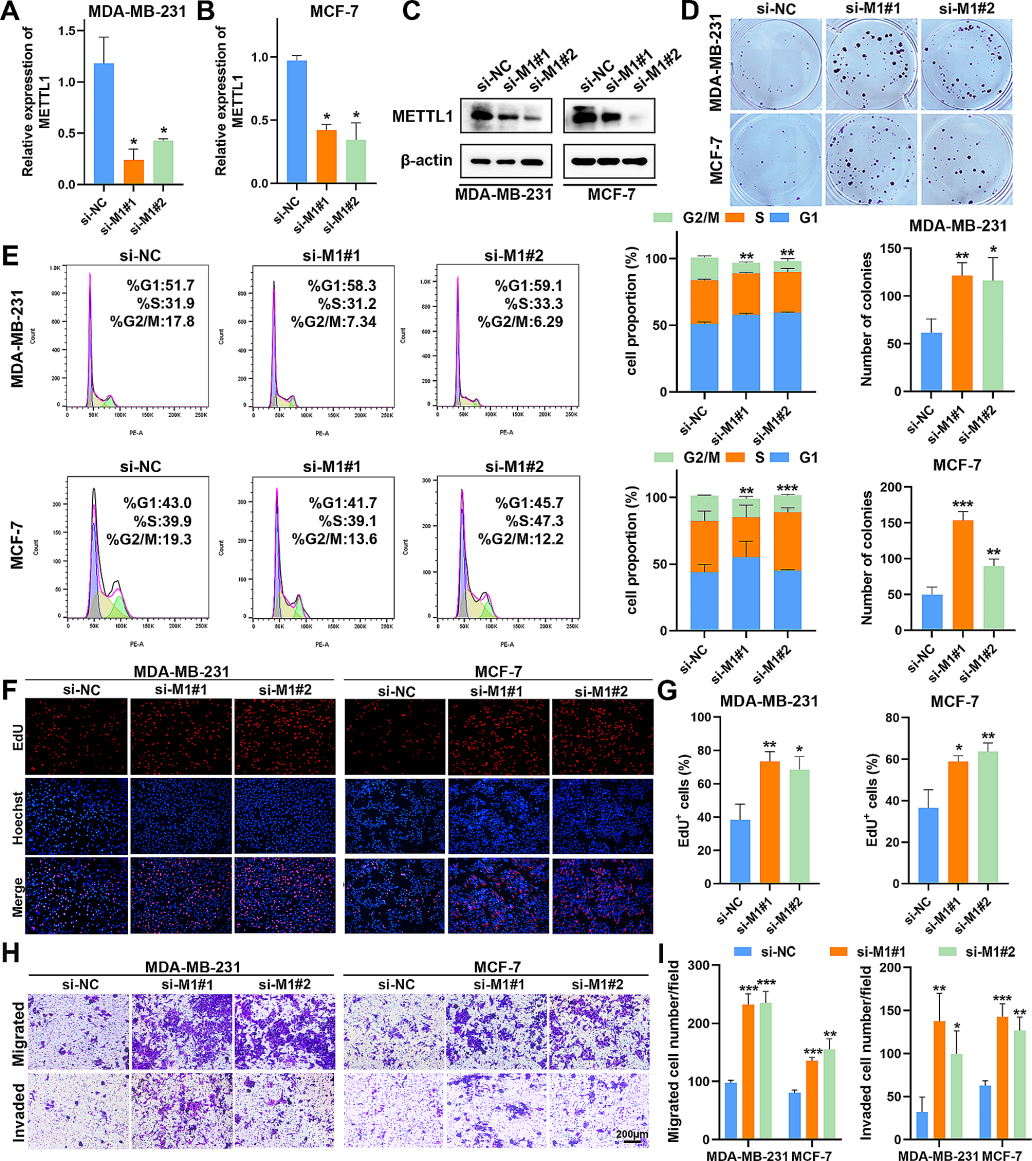
Inhibition of METTL1 promotes BC progression in vitro. (**A**-**B**) qRT-PCR analysis of relative METTL1 expression after METTL1 knockdown in MDA-MB-231 and MCF-7 cells. (**C**) Verification of METTL1 knockdown by western blotting in MDA-MB-231 and MCF-7 cells. (**D**) Colony formation assays assessing the clonogenicity of MDA-MB-231 and MCF-7 cells upon METTL1 silencing. (**E**) Flow cytometric analysis of cell cycle distribution. (**F**-**G**) EdU assays to evaluate cell proliferation. (**H**-**I**) Transwell assays to analyze cell migration and invasion ability. Scale bar = 200 μm. The data are presented as mean ± SD. *P < 0.05, **P < 0.01, ***P < 0.001

The METTL1/WDR4 complex plays a crucial role in mediating m^7^G methylation modifications on tRNAs, with METTL1 primarily responsible and WDR4 acting as a cofactor [20]. Given this, we focused our investigation on the impact of METTL1 on BC carcinogenesis. To achieve this, we employed two siRNAs, targeting METTL1 (si-M1#1 and si-M1#2), to knock down METTL1 expression in MDA-MB-231 and MCF-7 cells (Fig. 2A-C). Our experimental results revealed that METTL1 knockdown led to increased proliferation and colony-forming abilities in both MDA-MB-231 and MCF-7 cells (Fig. 2D; Supplementary Fig. S2A). Flow cytometry analysis demonstrated a significant reduction in the proportion of G2/M phase cells upon METTL1 deficiency (Fig. 2E). To explore the effects of METTL1 on BC cell proliferation and apoptosis, we conducted EdU and TUNEL assays, respectively. EdU immunofluorescence staining revealed that METTL1 knockdown substantially accelerated the proliferation of MDA-MB-231 and MCF-7 cells (Fig. 2F-G). Furthermore, the silencing of METTL1 markedly decreased the apoptosis of BC cells (Supplementary Fig. S2D-E). Transwell assays were performed to assess alterations in migration and invasion abilities, revealing a significant increase in the number of migratory and invasive cells in the si-METTL1 groups (Fig. 2H-I). Additionally, wound-healing assays demonstrated that METTL1 depletion enhanced the migration of MDA-MB-231 and MCF-7 cells (Supplementary Fig. S2B-C). Taken together, these observations offer compelling evidence supporting the tumor-suppressor function of METTL1 in BC tumorigenesis.

### Overexpression of wild-type METTL1 but not its catalytically inactive mutant impairs BC progression

**Fig. 3:**
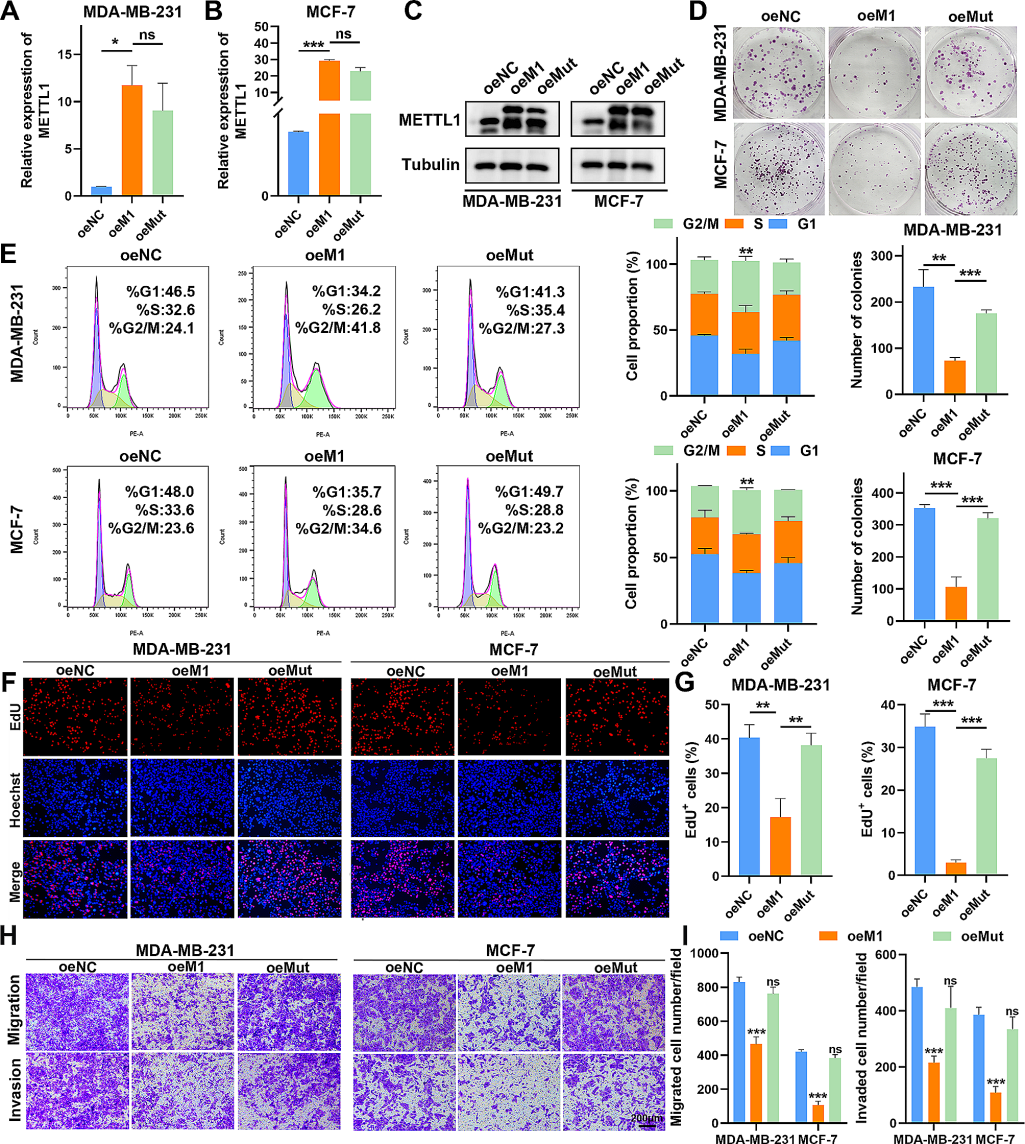
Overexpression of METTL1 impedes BC progression in vitro. (**A**-**B**) qRT-PCR analysis of relative METTL1 levels after forced expression of wild-type and catalytically inactive METTL1 in MDA-MB-231 and MCF-7 cells. (**C**) Western blotting confirmation of METTL1 in indicated MDA-MB-231 and MCF-7 cells. (**D**) Colony formation assays to assess the effects of METTL1 overexpression on the clonogenicity of MDA-MB-231 and MCF-7 cells. (**E**) Flow cytometric analysis of cell cycle distribution. (**F**-**G**) EdU assays to evaluate changes in BC cell proliferation. (**H**-**I**) Transwell assays to profile cell migration and invasion capacity. Scale bar = 200 μm. The data are presented as mean ± SD. *P < 0.05, **P < 0.01, ***P < 0.001, and ns., not significant

To further validate the impact of METTL1 on BC progression, we conducted gain-of-function studies in BC cells. Lentivirus vectors were utilized to overexpress wild-type METTL1 and its catalytically inactive mutant (aa160-163, LFPD to AFPA) (Fig. 3A-C). Functionally, the overexpression of wild-type METTL1 noticeably inhibited the proliferation and colony-forming capacities of BC cells (Fig. 3D; Supplementary Fig. S3A). Furthermore, flow cytometry analysis revealed that MDA-MB-231 and MCF-7 cells were arrested in the G2/M phase after wild-type METTL1 overexpression (Fig. 3E). The outcomes demonstrated that the exogenous expression of wild-type METTL1 suppressed the growth, migration, and invasion capacities of both MDA-MB-231 and MCF-7 cells (Fig. 3F-I; Supplementary Fig. S3B-C). Additionally, ectopic expression of wild-type METTL1 significantly enhanced apoptosis in MDA-MB-231 and MCF-7 cells (Supplementary Fig. S3D-E). However, overexpression of the catalytically inactive mutant METTL1 (oeMut) had little effect on these malignant behaviors of BC cells (Fig. 3D-I; Supplementary Fig. S3A-E). These findings strongly suggest that the m^7^G tRNA methyltransferase activity of METTL1 is essential for its regulatory function in BC transformation.

### METTL1 controls m^7^G tRNA modification and global mRNA translation in BC

**Fig. 4:**
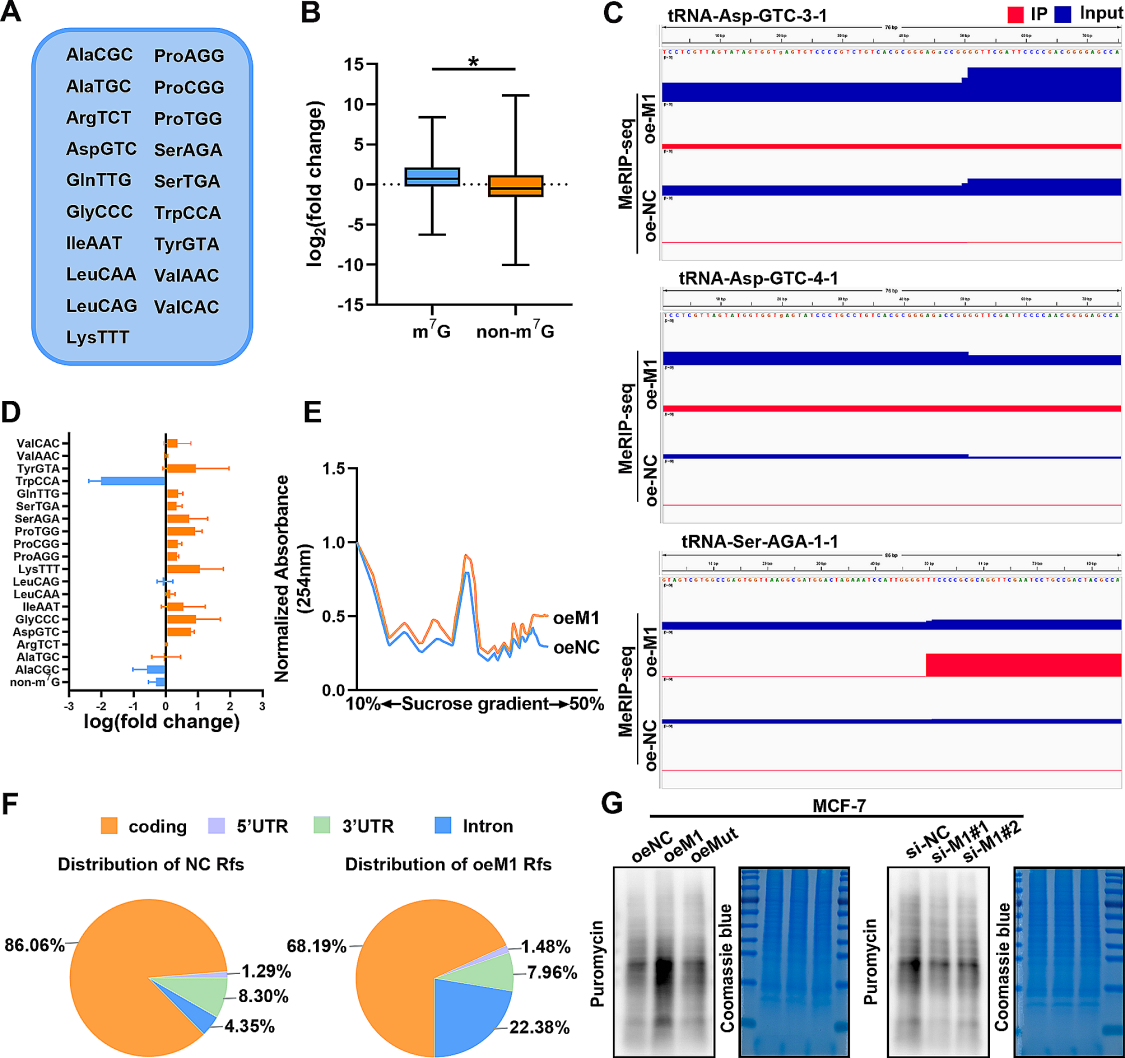
METTL1 enhances m^7^G tRNA methylation levels and global mRNA translation. (**A**) List of m^7^G-modified tRNAs identified by m^7^G tRNA MeRIP-seq in MCF-7 cells. (**B**) Quantitative comparison of fold change in expression between m^7^G and non-m^7^G tRNAs. (**C**) Representative images from Integrative genome viewer (IGV) displaying different IP/Input at the motif sequence of indicated tRNA. (**D**) Relative expression profile of m^7^G-modified tRNAs based on m^7^G tRNA MeRIP-seq. The relative expression of each tRNA type was calculated by combining the expression of all tRNA genes belonging to the same tRNA type. (**E**) Polysome profiling of MCF-7 cells with or without METTL1 overexpression. (**F**) Distribution of ribosome-protected fragments (Rfs). (**G**) Measurement of puromycin intake in MCF-7 cells overexpressing wild-type or mutant METTL1 compared to control, and in METTL1-depleted MCF-7 cells compared to control. Coomassie brilliant blue staining of the gel was used as a control. The data are presented as mean ± SD. *P < 0.05

Since METTL1 has been confirmed as a tRNA m^7^G methyltransferase, we investigated the relationship between METTL1-mediated m^7^G modification and tRNA levels in BC. To accomplish this, we performed m^7^G tRNA MeRIP-Seq in MCF-7 cells. Our data revealed 19 tRNAs with m^7^G modifications, including AspGTC and SerAGA (Fig. 4A). Additionally, m^7^G tRNA MeRIP-seq demonstrated that overexpression of METTL1 increased the expression levels of most m^7^G-modified tRNAs, while non-m^7^G-modified tRNAs remained largely unaffected (Fig. 4B). The elevated m^7^G signal in the m^7^G-modified tRNAs further supported the effect of METTL1 overexpression (Fig. 4C). Analysis of tRNA expression from m^7^G tRNA MeRIP-seq indicated that ectopic expression of METTL1 elevated the abundance of most m^7^G-modified tRNAs (Fig. 4D). Considering the correlation between tRNA abundance and protein synthesis, it is reasonable to speculate that METTL1 may influence mRNA translation. Therefore, we conducted polysome profiling to explore the role of m^7^G tRNA modifications in mRNA translation regulation. The results revealed an increase in the polyribosome peak upon METTL1 overexpression (Fig. 4E). Ribo-seq confirmed the presence of ribosome-protected fragments primarily located in the coding DNA sequence, as expected (Fig. 4F). Furthermore, puromycin intake assays provided additional confirmation that METTL1 overexpression promoted new protein synthesis in MCF-7 cells. Conversely, forced expression of the catalytically inactive mutant METTL1 had minimal impact on mRNA translation. METTL1 depletion led to a decrease in puromycin-labeled newly synthesized protein in MCF-7 cells (Fig. 4G). These findings strongly support the essential role of METTL1’s m^7^G catalytic function in promoting overall mRNA translation. In summary, our data demonstrate the critical involvement of METTL1-mediated m^7^G tRNA modification in tRNA expression and mRNA translation in BC.

### METTL1 regulates the translation of anti-oncogenic mRNAs in m^7^G tRNA decoded codon-dependent manner

**Fig. 5:**
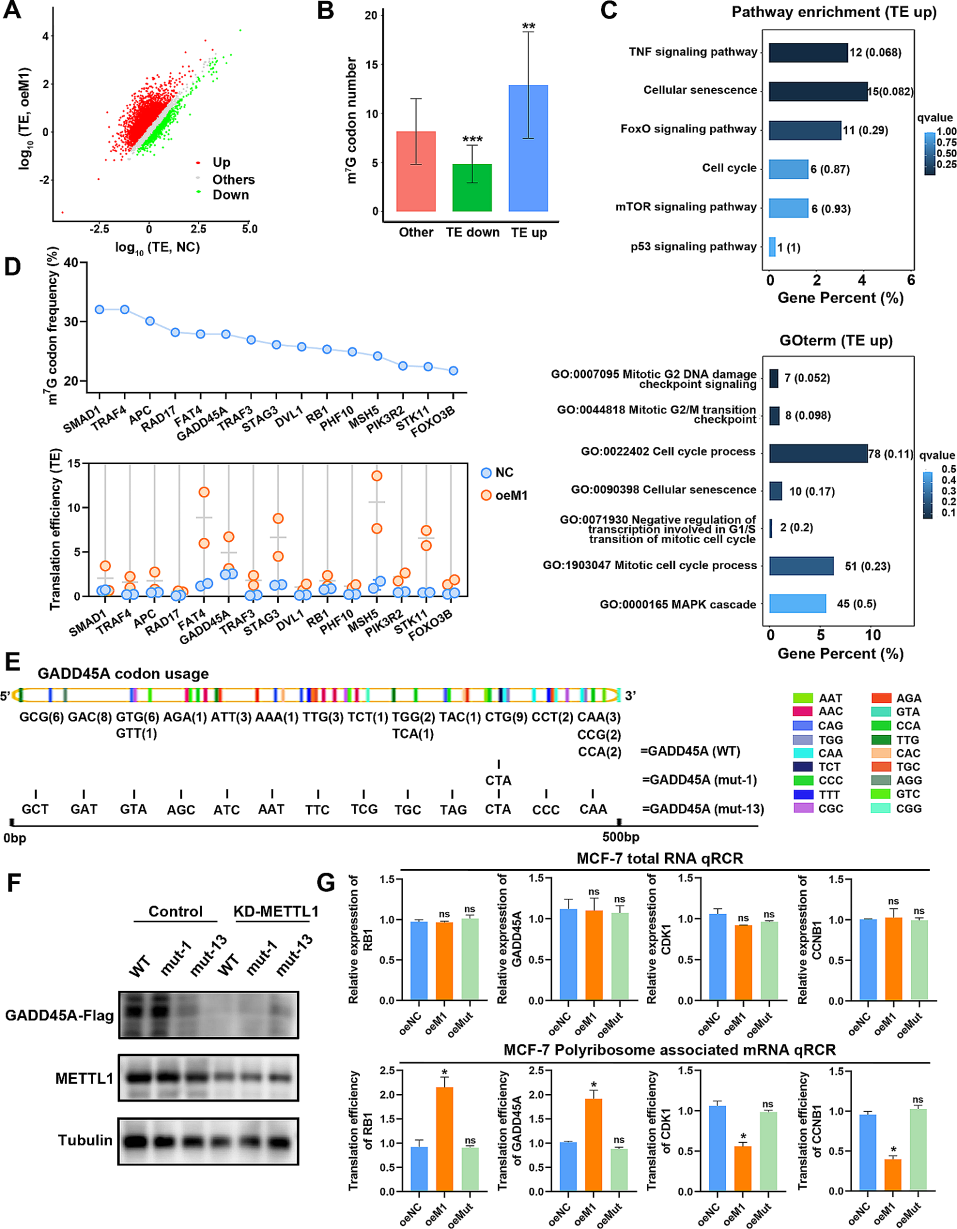
METTL1-mediated m^7^G tRNA modification selectively modulates the translational efficiency of specific transcripts. (**A**) Scatterplot of TE in MCF-7 cells with or without METTL1 overexpression. TE was calculated as the ratio of polyribosome signals to input signals. (**B**) Frequencies of m^7^G-related codons in TE-increased genes (TE-up), TE-decreased genes (TE-down), and other genes (others) in METTL1-overexpressing cells. (**C**) Pathway analysis (upper panel) and Gene Ontology analysis (lower panel) of TE-up genes upon METTL1 overexpression. (**D**) Frequency analysis of the 19 m^7^G-modified tRNAs decoded codons in 15 mRNAs associated with negative regulation of the cell cycle. (**E**-**F**) Western blotting analysis of GADD45A protein expression in 293T control and METTL1-knowdown cells overexpressing GADD45A WT-Flag, GADD45A mut-Flag (mutant 1 codons, CTA), or GADD45A mut-13-Flag (mutant 13 codons). (**G**) Relative expression and translation efficiency of RB1, GADD45A, CDK1, and CCNB1 mRNA in METTL1-overexpressed and control MCF-7 cells. Tubulin was used as an internal control. TE was calculated as the ratio of polyribosome signals to input signals. The data are presented as mean ± SD. *P < 0.05, **P < 0.01, ***P < 0.001, and ns., not significant

To investigate the downstream mRNA targets of METTL1-mediated m^7^G tRNA modification, we performed Ribo-seq and RNA sequencing (RNA-seq) in both METTL1 overexpressed and control MCF-7 cells. Our findings revealed significant alterations in TEs due to METTL1 overexpression, with an increase observed in the TEs of numerous mRNAs (Fig. 5A). Notably, codon frequency analysis indicated that mRNAs exhibiting higher TEs in METTL1 overexpressing cells possessed larger codons decoded by m^7^G-modified tRNAs (Fig. 5B). Furthermore, pathway analysis based on Ribo-seq data showed that the upregulated translated mRNAs in METTL1 overexpressed cells were strongly associated with the cell cycle processes as well as cellular senescence, thereby confirming the observed changes in the distribution of METTL1 overexpressing cells throughout the cell cycle. Consistently, Gene Ontology (GO) enrichment analyses demonstrated an association between genes with increased TEs and cell cycle processes, and that cell cycle effects were more pronounced in the regulation for the G2/M phase than in the G1/S phase (Fig. 5C). It is worth noting that mRNAs related to cell cycle processes that exhibited increased TEs were enriched in the mitotic G2/M transition checkpoint and negative regulation of the G1/S phase of mitosis. Of particular interest among these related mRNAs was GADD45A, which displayed higher TE and greater occupancy of m^7^G-related codons (Fig. 5D). GADD45A and RB1 are well-established cell cycle regulatory proteins responsible for G2/M phase arrest and G1/S phase arrest, respectively. Therefore, we next turned to investigating GADD45A and RB1 mRNAs in further research. Firstly, to investigate whether METTL1 regulates GADD45A mRNA translation through modification of m^7^G tRNA, we generated cDNAs of GADD45A mutants. In one mutant variant, we replaced codon GTG with CTA to generate GADD45A (mut-1), while in the other mutant variant, we replaced all m^7^G tRNA-decoded codons with their non-m^7^G tRNA decoded synonymous counterparts to generate GADD45A (mut-13) (Fig. 5E). Upon transient transfection of equal amounts of WT or mutant GADD45A cDNAs (mut-1 and mut-13) into 293T cells, western blotting analysis demonstrated that GADD45A expression was solely influenced when transfected with mut-13. In contrast, no significant changes were observed between WT and mutant GADD45A in METTL1-knockdown 293T cells (Fig. 5F). In addition, we conducted a polyribosome-associated mRNA-qPCR assay, which confirmed that the TEs of GADD45A and RB1 mRNAs were increased, while their mRNA levels remained unchanged in cells overexpressing METTL1. Notably, it is known that GADD45A plays an essential role in mediating cell cycle arrest at G2/M by interacting with CDK1 and Cyclin B1 (CCNB1) [23]. Given the elevated expression of GADD45A due to METTL1 overexpression, we questioned whether overexpressing METTL1 would also affect the translation efficiencies of CDK1 and CCNB1. Surprisingly, our results indicated that METTL1 overexpression decreased the TEs of both Cyclin B1 and CDK1 mRNA, with minimal impact on the mRNA levels of these transcripts (Fig. 5G). Collectively, our data reveal that the overexpression of METTL1 can impede the progression of BC cancer by regulating m^7^G-mediated up-regulation of tRNA, thereby enhancing the translation of GADD45A.

### METTL1-mediated phenotypic changes are associated with the translation of GADD45A

**Fig. 6:**
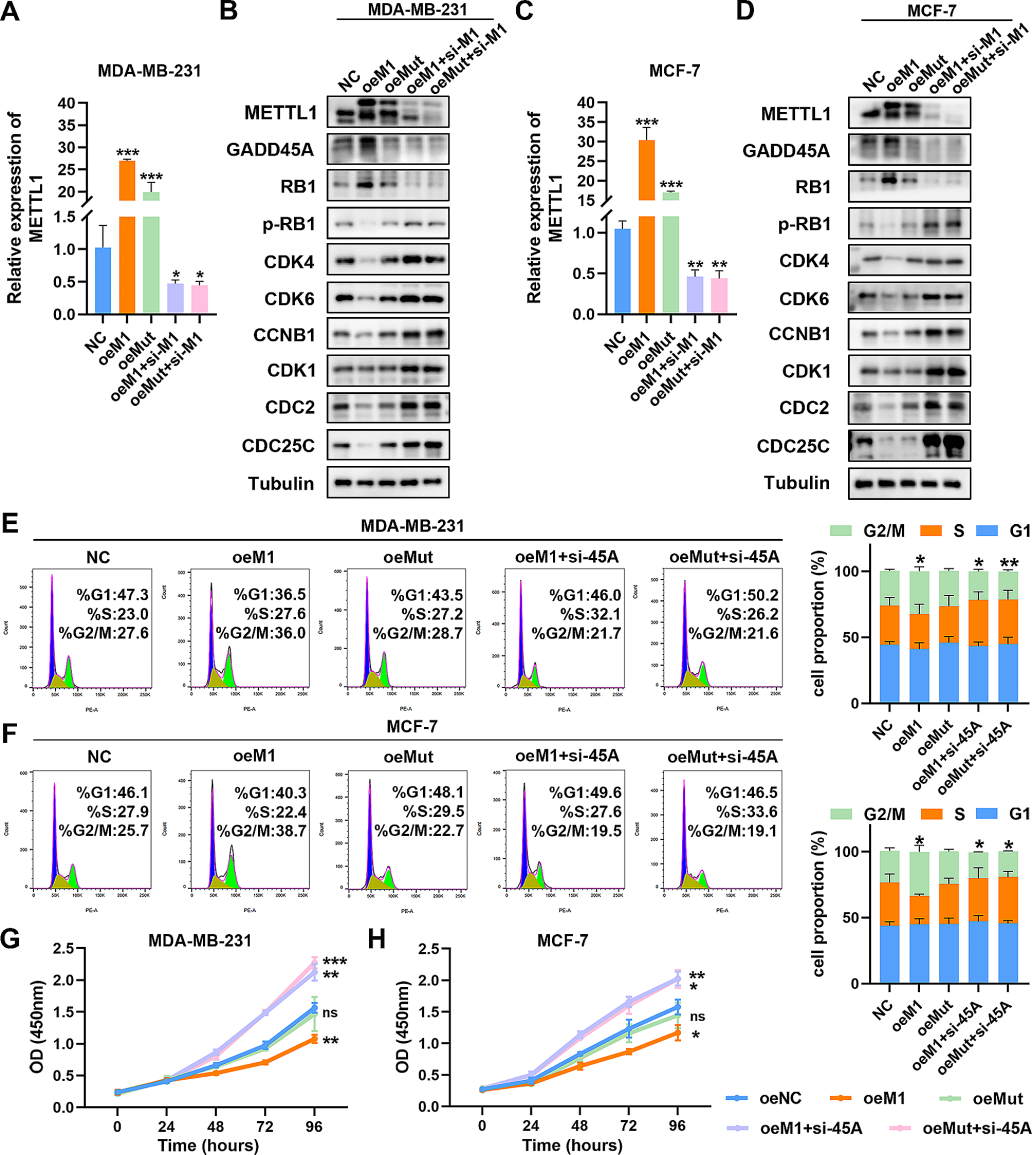
METTL1 controls BC malignant phenotypes through GADD45A. (**A**) qRT-PCR analysis of relative METTL1 expression after forced expression of wild-type METTL1 (oeM1) and catalytically inactive METTL1 (oeMut), followed by knockdown of METTL1 (oeM1 + si-M1, oeMut + si-M1) in MDA-MB-231 cells. (**B**) Western blotting analysis of GADD45A, RB1, and their downstream signaling molecules in different treatment groups of MDA-MB-231. (**C**) qRT-PCR analysis of relative METTL1 expression in oeM1, oeMut, oeM1 + si-M1, and oeMut + si-M1 in MCF-7 cells. (**D**) Western blotting analysis of GADD45A, RB1, and their downstream signaling molecules in different treatment groups of MCF-7. (**E**-**F**) Flow cytometry analysis of cell cycle phase distribution in MDA-MB-231 and MCF-7 treatment groups (oeM1, oeMut, oeM1 + si-45 A, oeMut + si-45 A) and control group (NC). (**G**-**H**) Assessment of proliferation ability using CCK-8 assays in MDA-MB-231 and MCF-7 cells. The data are presented as mean ± SD. *P < 0.05, **P < 0.01, ***P < 0.001, and ns., not significant

Next, we conducted loss- and gain-of-function assays to investigate the association between METTL1-induced phenotypic changes in BC cells and the translation of GADD45A. Consistently, our qRT-PCR assays revealed a distinct elevation in METTL1 levels in MDA-MB-231 and MCF-7 cells upon METTL1 overexpression, while siRNA-mediated depletion of METTL1 resulted in reduced METTL1 levels in oeMETTL1 and oeMut cells (Fig. 6A and C). Additionally, we examined the expression of downstream proteins and observed an upregulation of GADD45A and RB1 upon METTL1 overexpression, which decreased in the si-METTL1 group. Notably, the expression levels of p-RB1 (Ser807/811) and related CDKs molecules exhibited an opposite trend to METTL1, such as CDK4/6, CDK1, CCNB1, CDC2, and CDC25C (Fig. 6B and D). The phenotyping experiments yielded results that aligned with the expression levels of cell cycle-related proteins. Specifically, G2/M phase arrest was more pronounced in MDA-MB-231 and MCF-7 cells following overexpression of WT-METTL1, but not mut-METTL1. Moreover, concurrent knockdown of GADD45A in oe-METTL1 and oe-Mut cells rescued the effects of G2/M phase arrest, as compared to the control group (Fig. 6C-F). Furthermore, the knockdown of GADD45A consistently attenuated the anti-cancer phenotypes mediated by METTL1 overexpression in BC, including cell proliferation, migration, invasion, and apoptosis (Fig. 6G-H; Supplementary Fig. S4A-I). In summary, these results validate GADD45A, a cell cycle-related protein, as a functionally essential target of METTL1 in the pathogenesis of BC.

### METTL1 synergistically enhances the anti-oncogenic effects of abemaciclib

**Fig. 7:**
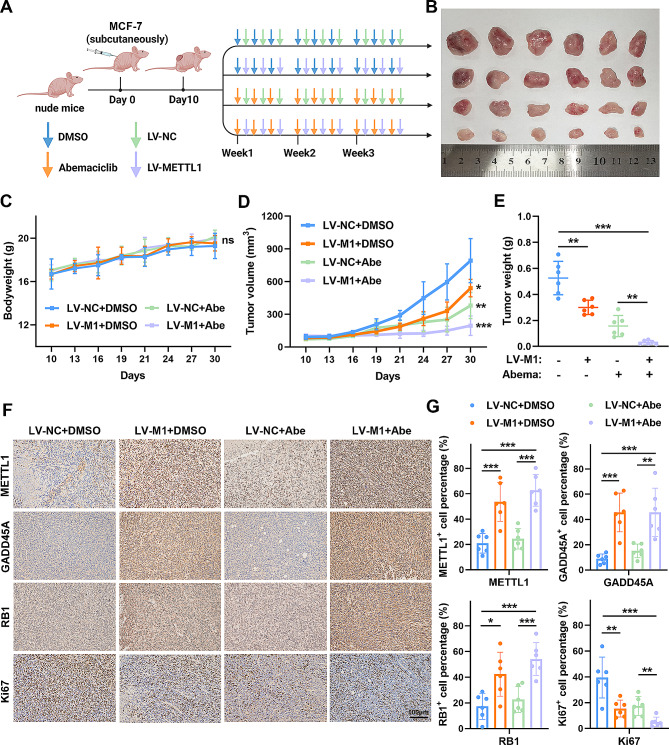
METTL1 enhances the anti-oncogenic effects of abemaciclib in vivo. (**A**) Schematic diagram to illustrate the treatment model protocol. (**B**) Representative images of tumors in xenograft mice. (**C**-**E**) Statistical analysis of body weight, tumor volume, and tumor weight in different groups of nude mice (*n* = 6). Data were measured every three days. (**F**) Representative IHC images of METTL1, GADD45A, RB1, and Ki67 expression in serial segments of tumor tissue separated from the subcutaneous model. (**G**) Quantification of METTL1, GADD45A, RB1, and Ki67 expression in xenograft tumor tissues. Scale bar = 100 μm. The data are presented as mean ± SD. *P < 0.05, **P < 0.01, ***P < 0.001, and ns., not significant

To further elucidate the role of METTL1 in vivo, we established xenograft tumor models using MCF-7 cells. Our data demonstrated that METTL1 overexpression significantly suppressed tumor growth compared to the control group (Supplementary Fig. S5A-C). IHC staining utilizing anti-METTL1 and anti-Ki67 antibodies confirmed the inhibitory effect of METTL1 on BC progression (Supplementary Fig. S5D-E). These findings suggest that METTL1 overexpression has the potential to impede the proliferation of BC cells in vivo. Based on our previous results indicating that METTL1 induces cell cycle arrest by promoting GADD45A and RB1 translation in BC, we assessed whether targeting METTL1 could enhance treatment efficacy when combined with abemaciclib, a dual CDK4/6 inhibitor approved for BC treatment. Mice bearing MCF-7 cells were injected with METTL1 lentivirus by multipoint intratumoral injection (LV-METTL1) and orally administered abemaciclib (three times per week) for a continuous 3-week period (Fig. 7A-B). None of the treatments-abemaciclib, LV-METTL1 alone, or their combination had noticeable effects on the mice’s weight (Fig. 7C). However, our data indicated that the combined treatment of abemaciclib with LV-METTL1 was more effective in inhibiting tumor growth compared to either treatment alone (Fig. 7D-E). IHC staining of Ki67 further revealed decreased proliferation in the combined treatment group relative to the other groups. Moreover, the expression of GADD45A and RB1 was increased upon forced expression of METTL1 in vivo (Fig. 7F-G). Our western blotting of subcutaneous xenografts in different treatment groups revealed that overexpression of METTL1 inhibited the expression of downstream associated CDKs, and the inhibitory effect was even more pronounced with the combination group (Supplementary Fig. S6A). Taken together, the in vivo results prove that METTL1 has the potential to potentiate the sensitivity to abemaciclib, thereby inhibiting BC tumor growth.

**Fig. 8:**
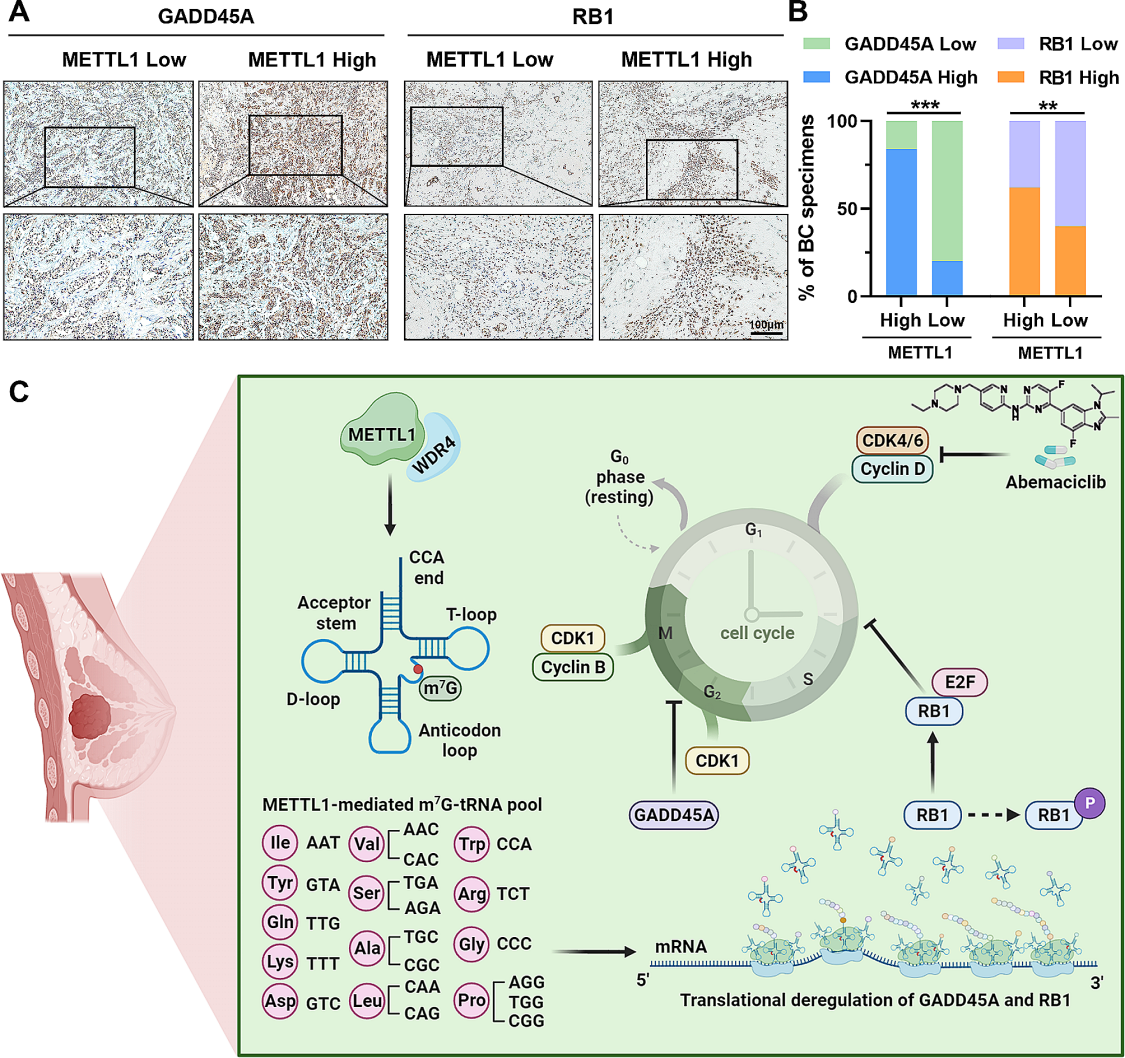
METTL1 positively correlates with GADD45A and RB1 expression in BC samples. (**A**-**B**) Representative images of IHC staining for GADD45A and RB1 expression in 50 BC samples with different levels of METTL1, Scale bar = 100 μm. (**C**) Schematic model to illustrate the role and potential mechanisms of m^7^G tRNA modification in regulating BC tumorigenesis and development. **P < 0.01, ***P < 0.001

At last, we explored the clinical relevance of METTL1, GADD45A and RB1 in the aforementioned BC patient’s cohort (Fig. 8A). Our analysis revealed a strong correlation between METTL1 and its downstream targets, GADD45A and RB1, as indicated by IHC staining (Fig. 8B). In conclusion, our findings demonstrate that METTL1 enhances the translation efficiency of GADD45A and RB1 in a manner dependent on m^7^G tRNA-decoded codons, leading to the inhibition of BC progression through G2/M phase arrest (Fig. 8C).

## Discussion

RNA modifications, such as N^6^-methyladenosine and m^7^G methylation, are emerging as key modulators of RNA biology and pathological processes [24]. These modifications can occur in both coding and non-coding RNAs, largely impacting RNA fate and activity [25]. Although recent studies have indicated the association between dysregulated RNA modifications and cancer, our understanding of the underlying molecular mechanisms is still in its early stages. The loss of m^7^G tRNA modification in yeast has been shown to result in temperature-sensitive growth at high temperatures, but the normal growth is unaffected, highlighting the importance of this modification in yeast’s response to environmental stress [26]. In contrast, the landscape of m^7^G modification in mammals is more intricate and widespread than in yeast. In mammals, the METTL1/WDR4 complex is responsible for installing m^7^G46 sites in tRNAs [27]. It has been shown that a series of conformational changes induced by METTL1 act as an activation switch for m^7^G methylation. Meanwhile, structural modeling points to an important role for the N-terminus of METTL1 in coordinating cofactor binding with major conformational alterations in the tRNA, the catalytic loop, and the C-terminal tail of WDR4, thus coordinating catalysis [28, 29]. Studies have revealed that m^7^G modification played a key role in maintaining pluripotency in human stem cells, and its deletion impairs self-renewal and neural differentiation of mouse embryonic stem cells [20, 30]. Furthermore, numerous recent studies have explored the relationship between m^7^G modification, METTL1/WDR4 complexes, and various diseases, particularly cancer. However, the expression pattern of m^7^G modification and METTL1 in BC are still poorly understood. Our findings showed lower expression of METTL1 and WDR4 in BC compared to normal tissues, at both mRNA and protein levels. However, we acknowledge the limitation of not elucidating the mechanism contributing to the downregulation of METTL1 expression in BC. A recent study identified a complex formed by histone acetyltransferase P300 and the transcription factor SP1, which binds to the METTL1 promoter region and modulates its transcriptional activation [31]. Another study lent support to the association between METTL1, androgen receptor, and Kallikrein-3 using specific datasets. It also suggested that the AKT/mTOR down-streamed signaling pathway regulated METTL1 expression, and elevated METTL1 promotes prostate cancer tumorigenesis through tRNA-derived fragment biogenesis [32]. Further investigations are necessary to uncover the potential upstream regulators responsible for modulating the low expression of METTL1 in BC.

METTL1 plays an influential part in the progression of various tumors, impacting tumor cell proliferation, autophagy, and sensitivity to radiotherapy and chemotherapy. Interestingly, our study revealed, for the first time, that METTL1 acted as a tumor suppressor in BC, which diverges from its role in most of the tumor types. Previous reports have shown that METTL1 promotes esophageal squamous cell carcinoma through the RPTOR/ULK1/autophagy axis and mediates m^7^G tRNA modification to promote lenvatinib resistance in hepatocellular carcinoma [33, 34]. Additionally, a study identifies METTL1 and m^7^G tRNA modification as selective regulators of mRNA translation, particularly impacting CDKs in intrahepatic cholangiocarcinoma [35]. However, Pandolfini et al. showed that METTL1 promotes microRNA (miRNA) maturation and inhibits the migration of lung cancer cells A549 [36]. Another study found that overexpression of METTL1 increases the sensitivity of colon cancer cells to cisplatin [37]. A recent study reported that METTL1 triggers osteosarcoma cells ferroptosis via the miR-26a/FTH1 axis which in turn induces iron death and increases the sensitivity of osteosarcoma cells to chemotherapeutic agents [38]. Our investigation, employing clinical patient samples, BC cell culture models, and xenograft models, compellingly demonstrated that METTL1 restrained BC tumorigenesis and progression both in vivo and in vitro. Through m^7^G tRNA MeRIP-seq and Ribo-seq analyses, we uncovered that METTL1-mediated m^7^G tRNA modification selectively regulates mRNA translation. Instead of directly influencing CDKs and Cyclins, METTL1 affected specific cell cycle inhibitors within the cell cycle pathway, including GADD45A and RB1, which play roles in inhibiting the progression of the G2/M phase and G1/S phase. Furthermore, our analysis of m^7^G tRNA occupancy and TE changes by Ribo-seq results indicated that GADD45A is more susceptible to METTL1 than RB1. As a result, BC cells overexpressing METTL1 experienced arrest predominantly in the G2/M phase. Collectively, our findings provided preliminary evidence for the mechanism underlying BC progression, whereby decreased METTL1 disrupts m^7^G-methylation mediated translational control of GADD45A and RB1, ultimately leading to BC progression.

GADD45 is a stress and DNA damage-responsive gene, initially identified for its induction following ultraviolet irradiation. It consists of three family members: GADD45A, GADD45B, and GADD45G. Among these, GADD45A serves as a tumor suppressor in several cancers and regulates cell cycle arrest, cell survival, and apoptosis in response to physiological and environmental stress [39]. Previous studies have demonstrated that GADD45A induces G2/S-phase cell cycle arrest through Cyclin B1, Cyclin D3, and CDC2 [23]. Additionally, GADD45A, induced by BRCA1, enhances apoptosis via activation of c-Jun N-terminal kinase/stress-activated protein kinase (JNK/SAPK) independently of p53 [40]. Furthermore, accumulating evidence suggests that the anti-cancer activities of chemotherapeutic agents and non-steroidal anti-inflammatory drugs depend on up-regulation of GADD45A, which induces cell cycle block and apoptosis in tumor cells [41, 42]. On the other hand, RB1 plays a crucial role in cell division by regulating E2F transcription factors, thereby controlling cell-cycle progression [17]. Mechanistically, METTL1 increases m^7^G methylation levels and tRNA expression, including GTA and AGA, resulting in elevated overall translation levels within the cell. Genes with increased translation efficiency undergo pathway analysis, revealing their association with cycle-related pathways. Notably, GADD45A mRNA in this pathway contains abundant m^7^G-related codons, pointing to a closer relationship with METTL1 and leading to a more pronounced elevation in translation efficiency. Consistent with previous experiments, we performed western blotting using a GADD45A mutant, confirming that METTL1 regulates GADD45A translation in an m^7^G-tRNA-dependent manner. Flow cytometry analysis also supports this, as METTL1 overexpression leads to G2/M phase cell cycle block in BC cells.

HR-positive and HER2-negative tumors are the most prevalent subtype of BC, accounting for approximately 70% of new cases [43]. The monarchE clinical trial provides evidence that CDK4/6 inhibitors significantly reduce the risk of disease recurrence or metastasis in HR-positive HER2-negative high-risk early-stage BC patients [44]. These observations underscore the pivotal role of CDK4/6 inhibitors in endocrine therapy for luminal-type BC patients. However, challenges regarding drug resistance and precision therapies remain for CDK4/6 inhibitors [45]. In our in vivo experiments, combining a lentivirus targeting METTL1 with CDK4/6 inhibitors demonstrated both safety and commendable therapeutic efficacy. CDK4/6 inhibitors target the RB1 tumor suppressor protein, causing G1/S phase cell cycle arrest, while METTL1 overexpression blocks the G2/M phase. Thus, the combination of METTL1 and abemaciclib inhibits tumor growth through a two-pronged approach. Our work suggest that developing agonists for METTL1 may represent a favorable therapeutic strategy to enhance the efficiency of CDK4/6 inhibitors in treating BC.

## Conclusion

Our study demonstrates the significant involvement of METTL1-mediated m^7^G tRNA modification in the development of BC. This research provides novel insights into the modulation of gene expression and codon preference through m^7^G-tRNA modification. We have presented compelling evidence supporting the crucial physiological role of METTL1 in BC development, highlighting its potential as a therapeutic candidate for BC treatment. Furthermore, our results support the combination of abemaciclib with METTL1 overexpression as a potential approach for BC therapy, offering valuable prospects for the future development of targeted drugs in this field.

### Electronic supplementary material

Below is the link to the electronic supplementary material.

Supplementary Material 1

## Data Availability

All data relevant to the study are included in the article or provided as supplementary information. The data that supporting the findings of this study are available from the corresponding author upon reasonable request.

## Abbreviations

tRNAs: transfer RNAs
BC: Breast cancer
m^7^G: N^7^-methylguanosine
METTL1: methyltransferase-like 1
WDR4: WD repeat domain 4
GADD45A: growth arrest and DNA damage 45 alpha
RB1: retinoblastoma protein 1
CDKs: cyclin-dependent kinases
CCNB1: Cyclin B1
HR+: hormone receptor-positive
HER2-: human epidermal growth factor receptor 2-negative
siRNA: Small interfering RNA
IHC: immunohistochemistry
CCK-8: Cell Counting Kit-8
TUNEL: TdT-mediated dUTP Nick-End labeling staining
ROC: Receiver operating characteristic curves
Rfs: ribosomal footprints
TEs: translation efficiencies
Ribo-seq: Ribosome profiling sequencing
m^7^G tRNA MeRIP-seq: m^7^G-methylated tRNA immunoprecipitation sequencing
