# Supplementary material for: METTL1-mediated tRNA m7G methylation and translational dysfunction restricts breast cancer tumorigenesis by fueling cell cycle blockade
Source: J Exp Clin Cancer Res. 2024 May 31;43:154. doi: 10.1186/s13046-024-03076-x (PMC11140866; doi:10.1186/s13046-024-03076-x)
Supplement: Supplementary file 1 — Supplementary Material 1 [file 13046_2024_3076_MOESM1_ESM.pdf]

Table S1. Baseline information of patients with breast cancer.

Characteristic	Number
**Age (mean±SD)**	50.1±8.5
**T stage, n (%)**	
T1	14 (21.21%)
T2	44 (66.67%)
T3	8 (12.12%)
T4	0 (0%)
**N stage, n (%)**	
N0	33 (50%)
N1	21 (31.82%)
N2	5 (7.58%)
N3	7 (10.61%)
**Pathologic stage, n (%)**	
Stage I	9 (13.64%)
Stage II	44 (66.67%)
Stage III	13 (19.70%)
Stage IV	0 (0%)
**ER status, n (%)**	
Negative	31 (46.97%)
Positive	35 (53.03%)
**PR status, n (%)**	
Negative	39 (59.09%)
Positive	27 (40.91%)
**HER2+ status, n (%)**	
Negative	18 (27.27%)
Positive	48 (72.73%)

Table S2. Sequences of primers used for qRT-PCR in this study.

Name	Sequence
METTL1 Forward	GGCAACGTGCTCACTCCAA
METTL1 Reverse	CACAGCCTATGTCTGCAAACT
WDR4 Forward	ACAGCCCTGACTTTCATAGCC
WDR4 Reverse	TCACAGCCACATCTAACAGCATA
RB1 Forward	TTGGATCACAGCGATACAAACTT
RB1 Reverse	AGCGCACGCCAATAAAGACAT
GADD45A Forward	GAGAGCAGAAGACCGAAAGGA
GADD45A Reverse	CACAACACCACGTTATCGGG
CDK1 Forward	AAACTACAGGTCAAGTGGTAGCC
CDK1 Reverse	TCCTGCATAAGCACATCCTGA
CCNB1 Forward	AATAAGGCGAAGATCAACATGGC
CCNB1 Reverse	TTTGTTACCAATGTCCCCAAGAG
GAPDH Forward	AAGGTGAAGGTCGGAGTCA
GAPDH Reverse	GGAAGATGGTGATGGGATTT
Tubulin Forward	TCCATGAAGGAGGTCGATGA
Tubulin Reverse	CAGACGGCTGTCTTGACATT

Table S3. Sequences of siRNA and shRNA used in this study.

Gene name	Probe (5'-3')
si-METTL1#1	GATGACCCAAAGGATAAGAAA
si-METTL1#2	GGATGTGCACTCATTTCGA
si-GADD45A	GGAGAGCAGAAGACCGAAA

Table S4. Primary antibodies.

Antibodies	Source	Identifier
Anti-METTL1	Proteintech	Cat#14994-1-AP
Anti-WDR4	Abcam	Cat#ab169526
Anti-β-actin	Cell Signaling Technology	Cat#13E5
Anti-Tubulin	Proteintech	Cat#66031-IG
Anti-puromycin	Kerafast	Cat#3RH11
Anti-GADD45A	Bioss	Cat#bs-1360R
Anti-RB1	Proteintech	Cat#10048-2-IG
Anti-p-RB1(Ser807/811)	Cell Signaling Technology	Cat#9308
Anti-CDK4	Bioss	Cat#bs-0633R
Anti-CDK6	Bioss	Cat#bs-0568R
Anti-CDK1	Bioss	Cat#bs-0542R
Anti-CCNB1	HuaBio	Cat#R1308-13
Anti-CDC2	Zenbio	Cat#200544
Anti-CDC25C	Zenbio	Cat#R381485
Anti-Ki67	ThermoFisher	Cat#14-5698-82

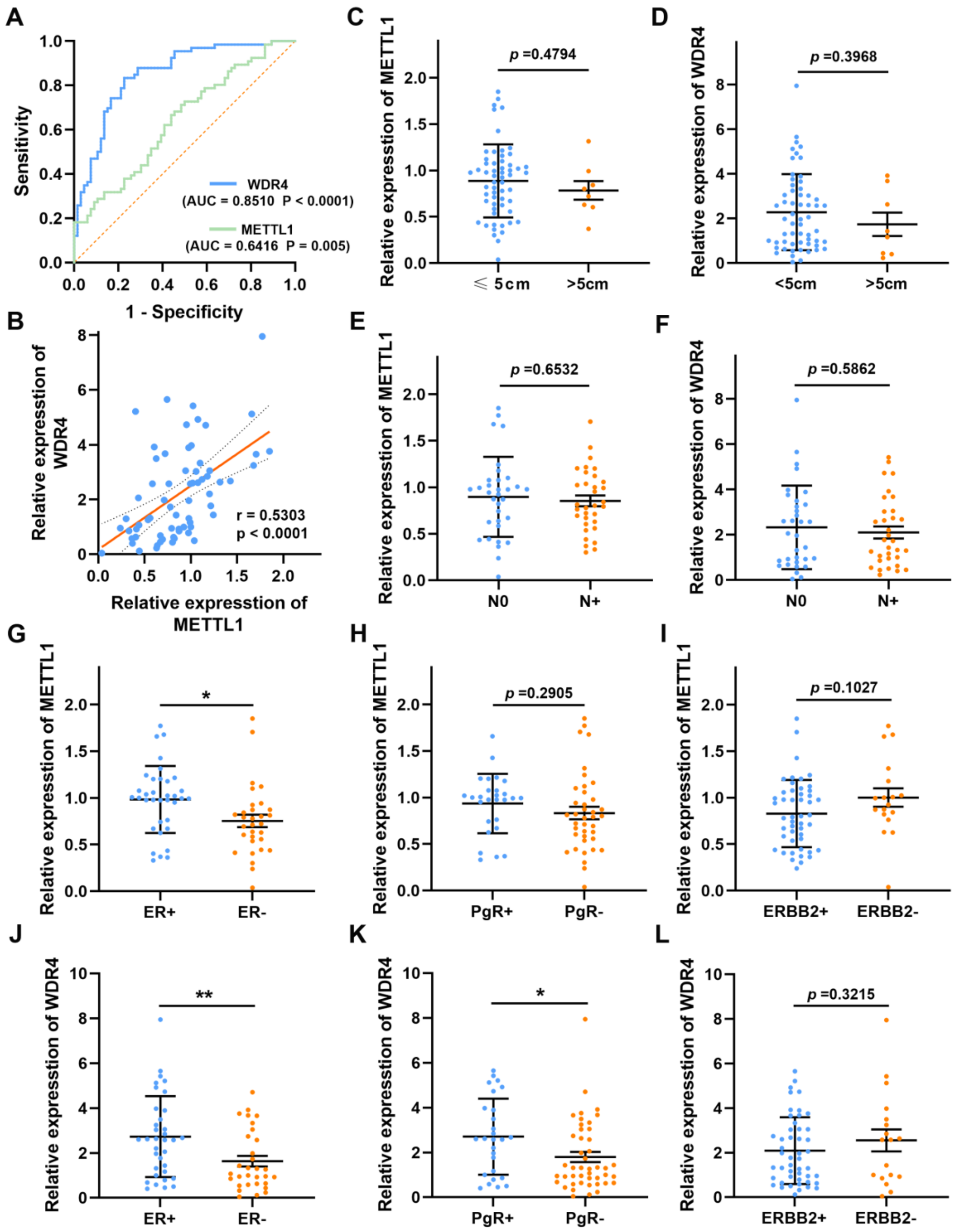

Figure S1: Association of METTL1 and WDR4 expression with clinicopathological variables in BC.

(A) ROC curve analysis of METTL1 and WDR4 expression in BC samples and adjacent normal breast tissue. (B) qRT-PCR analysis of mRNA correlation between METTL1 and WDR4 expression in a cohort of 66 BC patients. (C-D) Statistical

analysis of METTL1 expression levels in BC patients with different tumor sizes. (E-F) Correlation analysis between METTL1 and WDR4 expression and lymph node status in BC patients. (G-L) Correlation analysis of METTL1 and WDR4 expression with ER, PgR, and ERBB2 status in BC patients. The data are presented as mean ± SD. *$P < 0.05$, **$P < 0.01$.

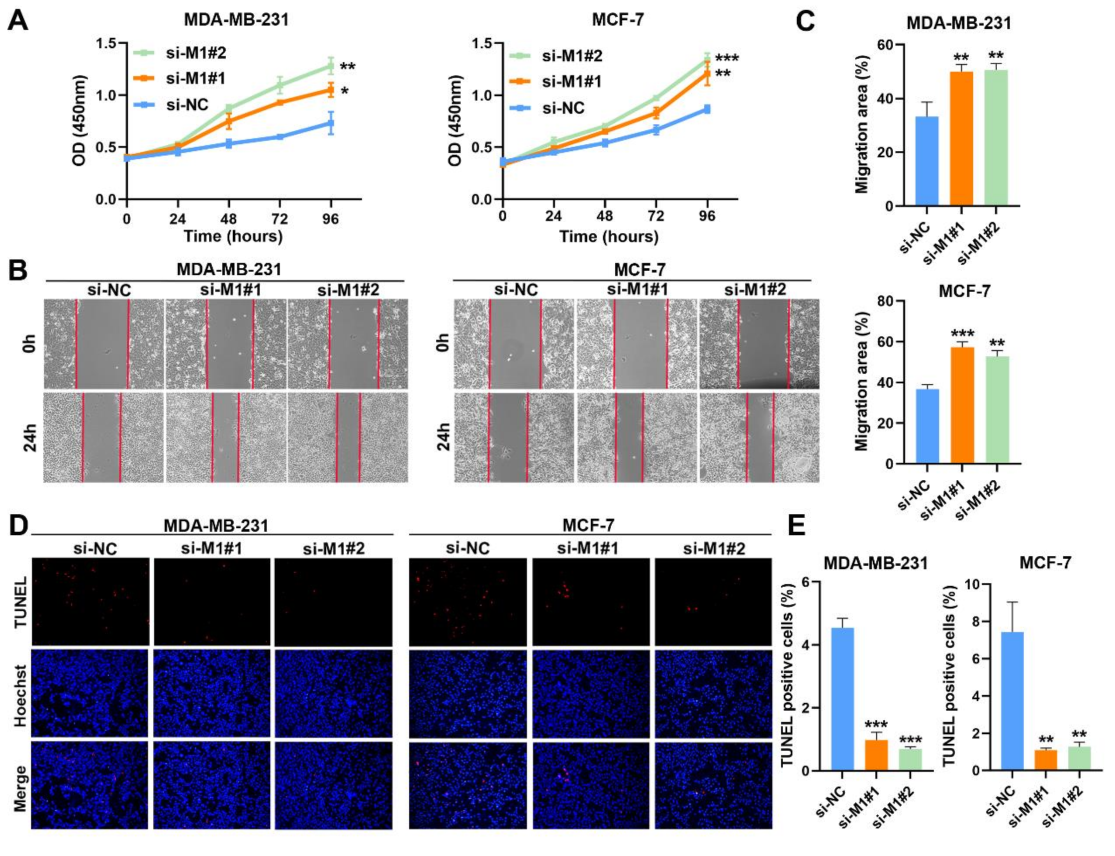

Figure S2: METTL1 is required for BC progression in vitro.

(A) Assessment of cell proliferative capacity using the CCK-8 assay in each group. (B-C) Evaluation of the effect of METTL1 knockdown on cell migration through wound healing assay. (D-E) TUNEL assay used to assess apoptotic ability of cells. The data are presented as mean ± SD. *P < 0.05, **P < 0.01, ***P < 0.001.

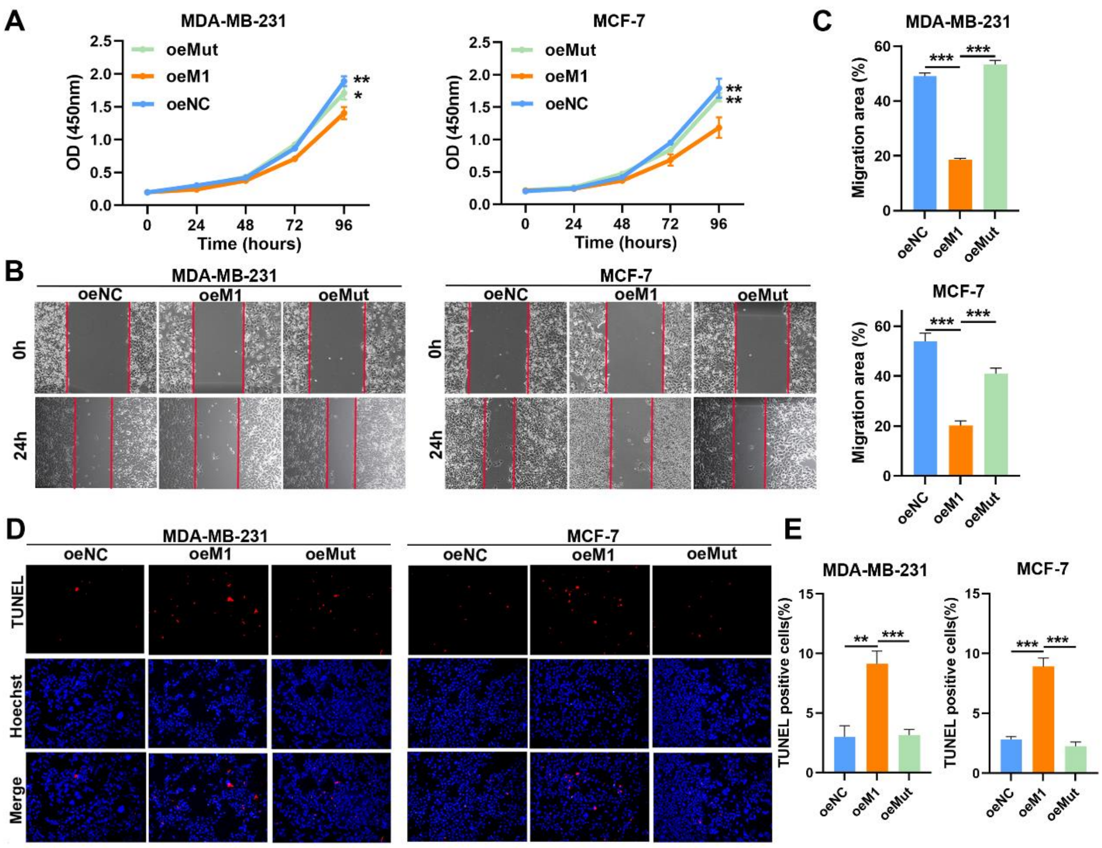

Figure S3: Overexpression of METTL1 restrains BC progression in vitro.

(A) CCK-8 assay measuring the effects of METTL1 activity on cell viability. (B-C) Investigation of migratory ability using wound healing assay in each group. (D-E) TUNEL assay to examine the apoptosis in each group of cells. The data are presented as mean ± SD. *P < 0.05, **P < 0.01, ***P < 0.001.

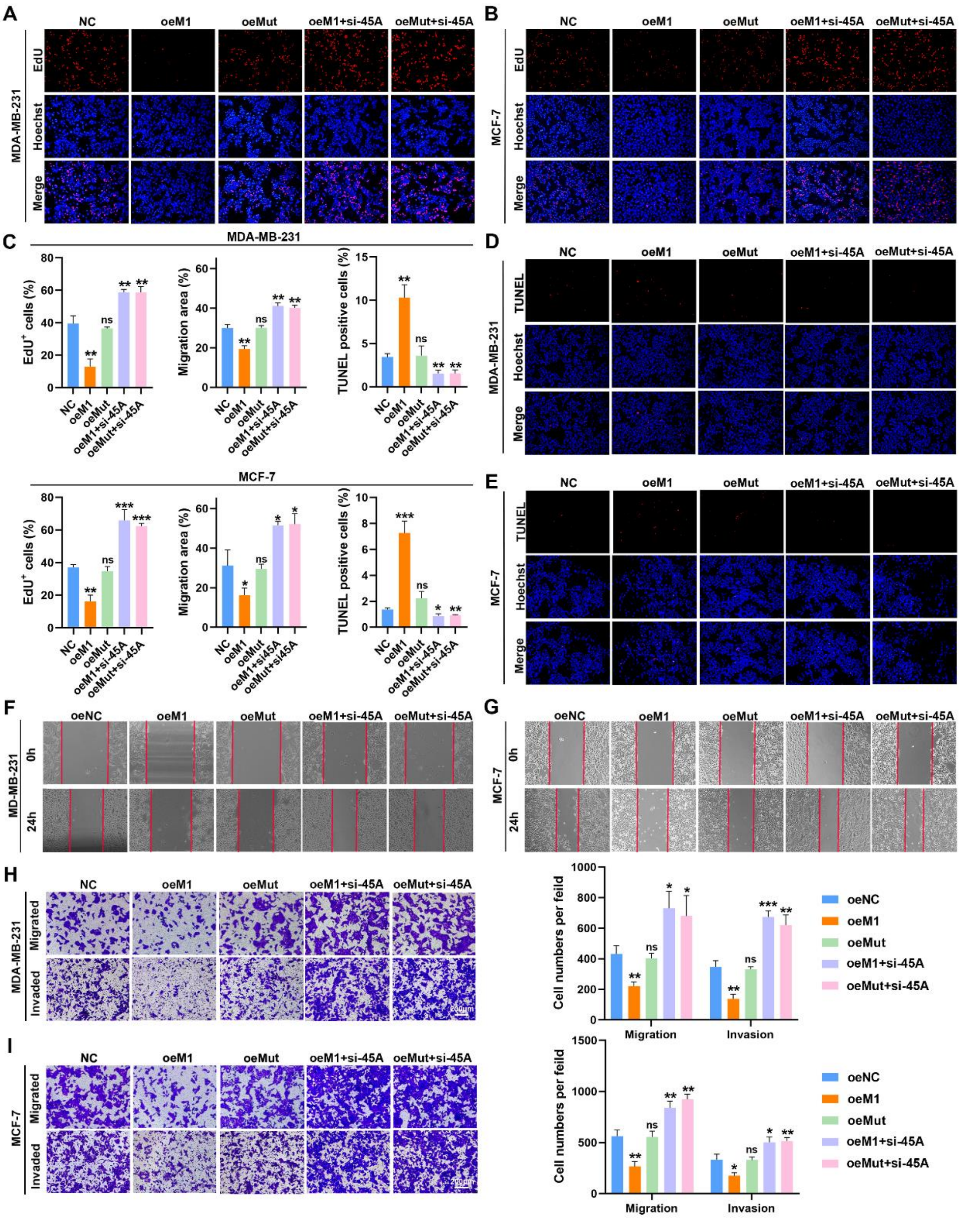

Figure S4: Knockdown of GADD45A reverses the inhibitory effect of METTL1 on BC.

(A-G) The proliferation, apoptosis and migration functions of the cells in different treatment groups (NC, oeM1, oeMut, oeM1+si-45A, oeMut+si-45A) of MDA-MB-231 and MCF-7 were evaluated ed by EdU assay (A-B), TUNEL assay (D-E) and wound

healing assay (F-G). The results of these experiments showed statistical differences (C). (H-I) Transwell assays to investigate cell migration and invasion ability in MCF-7 and MDA-MB-231 cells in different treatment groups, Scale bar = 200 μm. The data are presented as mean ± SD. *P < 0.05, **P < 0.01, ***P < 0.001, and ns., not significant.

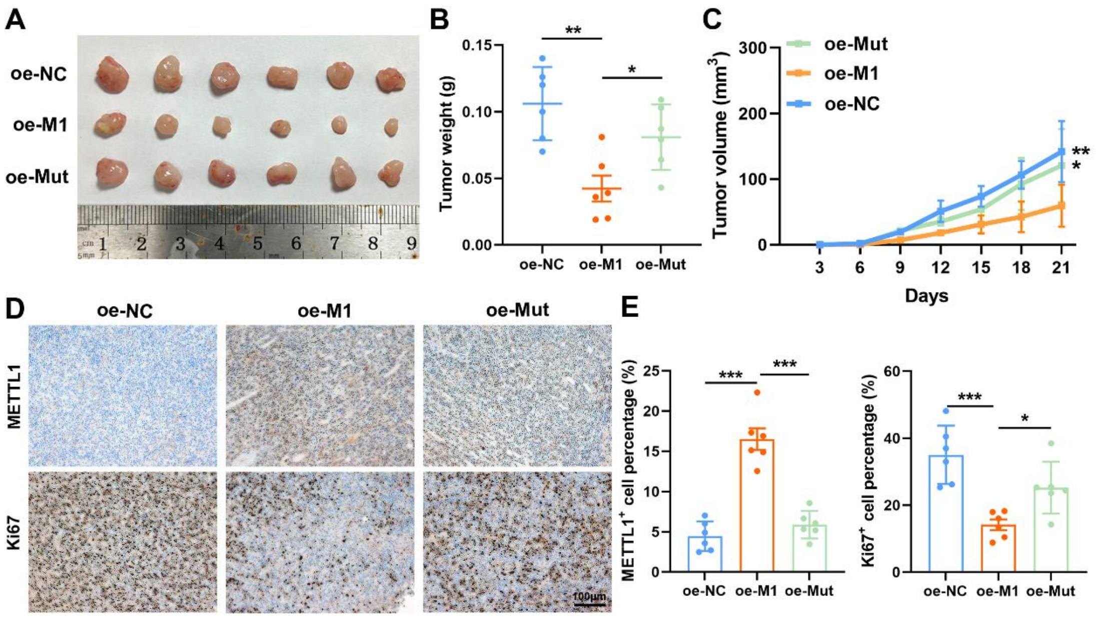

Figure S5: METTL1 overexpression inhibits BC growth in vivo.

(A) Representative images of tumors harvested from MCF7 xenografts in nude mice. (B-C) Analysis of tumor volume and tumor growth curves in xenograft mice. (D) Representative IHC images of subcutaneously implanted mouse models, Scale bar = 100 µm. (E) Quantification of METTL1 and Ki67 positive cells in the indicated groups. The data are presented as mean ± SD. *P < 0.05, **P < 0.01, ***P < 0.001.

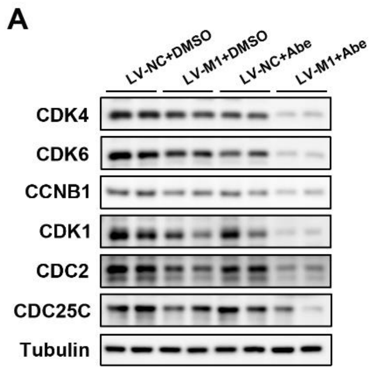

Figure S6: Combined METTL1 with abemaciclib attenuated the expression levels of related downstream cell cycle regulators in vivo.

(A) Western blotting was conducted to assess the expression levels of the cell cycle-related regulators CDKs, CCNB1, CDC2 and CDC25C in different groups of nude mice (n = 2 randomly selected from each group).
